# Analysis of hospital dental care for patients with special needs in Brazil

**DOI:** 10.1590/1807-3107bor-2024.vol38.0007

**Published:** 2024-05-10

**Authors:** Amanda Beatriz Gonçalves VIVACQUA, Edson Hilan Gomes de LUCENA, Gilberto Alfredo PUCCA JÚNIOR, Fábio Carneiro MARTINS

**Affiliations:** (a) Universidade de Brasília – UnB, School of Health Sciences, Department of Dentistry, Brasília, DF, Brazil.; (b) Universidade Federal da Paraíba – UFPB, Department of Clinical and Social Dentistry, João Pessoa, PB, Brazil.; (c) Universidade de Brasília – UnB, School of Health Sciences, Department of Oral Health, Brasília, DF, Brazil.; (d) Universidade de São Paulo – USP, School of Dentistry, Department of Social Dentistry, São Paulo, SP, Brazil.

**Keywords:** Sistema Único de Saúde, Dental Care, Disabled Persons, Health Services Accessibility

## Abstract

This analytical cross-sectional study aimed to analyze the access of patients with special needs (PSN) in Brazilian municipalities to hospital dental care of the Unified Health System (Sistema Único de Saúde - SUS), based on data from the Hospital Information System of the Unified Health System (*Sistema de Informações Hospitalares do SUS*- SIH/SUS - SIH), from 2010 to 2018. The Kolmogorov-Smirnov normality test was performed; the Poisson regression was used to verify factors associated with the variable total number of hospitalization authorizations with the main procedure of dental treatment for PSN (“Total de Autorizações de Internação Hospitalar” – AIH), the Spearman correlation test with a significance level of 5% was used to characterize the relationships between the Municipal Human Development Index per municipality - (*Índice de Desenvolvimento Humano Municipal* - HDI) and the Oral Health Coverage in the Family Health Strategy by municipality (*Cobertura de saúde bucal na estratégia saúde da família por município* - SBSF Coverage), and the relationship of the AIH with SBSF Coverage. A total of 127,691 procedures were performed, of which 71,517 (56%) were clinical procedures, such as restorations, endodontic treatments, supra and subgingival scaling, among others. Municipalities in the Midwest (PR=5.117) and Southeast (RP = 4.443) regions had more precedures than the others. A weak correlation was found between AIH and SBSF Coverage (r = -0.2, p < 0.001) and HDI and SBSF Coverage (r = -0.074, p < 0.001). Population size, region, health coverage, oral hygiene, and number of dentists in hospitals affected the availability of dental procedures in PSN.

## Introduction

Hospital Dentistry can be understood as a set of preventive, diagnostic, and therapeutic actions for orofacial diseases, oral manifestations of systemic origin, or sequelae of their respective treatments that require interventions by multidisciplinary teams in highly complex care settings.^1^

The health system in Brazil - the Unified National Health System (*Sistema Único de Saúde* - SUS) was developed during the 1980s as part of the social movement aimed at Brazil’s redemocratization, consisting of a set of health actions and services provided by federal public bodies and institutions, state and municipal levels and being based on principles and guidelines,^2^ promoting universalization, decentralization, equity, integrality, and social control of health in Brazil. This has made it the largest public health system in the world, with primary care reaching around 145 million Brazilians in 2020.^3^

In 2004, the National Oral Health Policy – “Brasil Sorridente” – emerged on the Brazilian scene as a counter-hegemonic model of attention to the existing dental practices in the country until then. This policy reoriented the model care with the implementation of a care network that articulates the three levels of care and multidisciplinary and intersectoral actions, in addition to the expansion and creation of new oral health services.^4^ With the creation of “Brasil Sorridente”, Brazil became a reference in oral health, being the largest public oral health program in the world, establishing the ideas that dental care should be provided by the Unified Health System at the national level in favor of universal access to comprehensive health services, contemplating all levels of care (comprehensiveness) with the installation of the Dental Specialty Centers (Centro de Especialidades Odontológicas- CEO), regional dental prosthetic laboratories, and the expansion of fluoridation coverage of the public water supply in Brazil, with the responsibility of developing the autonomy of the individual, encouraging participatory management, and assuming all health problems of the population.^5^ Although Brazil has admittedly made advancements by establishing universal and comprehensive care and increasing dental assistance, hospital dentistry still has little visibility. Despite this, in the context of the SUS, the care networks are an important reference axis by providing technical assistance activities carried out on an outpatient and inpatient basis, as routine or urgency in the secondary and tertiary health care.^1,6^

The term “people with special needs” (PSN – Indivíduos com Necessidades Especiais) in dentistry includes people with one or more temporary or permanent limitations of a mental, physical, sensory, emotional, growth or medical nature, which prevent them to undergo a conventional dental treatment.^7^ The presence of a dentist as a reference for the multidisciplinary team responsible for high-complexity (hospital) care is essential for comprehensive care for PSN, as it contributes to the care of users who need to attend this level of health care for clinical follow-up and specific treatment, which may require the provision of specific resources for safe esthetic and functional rehabilitation.^6^ Furthermore, it is known that oral health professionals play a fundamental role in the care of patients admitted to intensive care units, who have several local and systemic risk factors for the development of oral diseases, minimizing the risk of infection, thus improving the quality of life and decreasing the length of stay of these patients remain in the hospital.^8^

## Brazilian history and legislation of the implantation of Hospital Dentistry

There is no universal definition for hospital dentistry, but internationally, it includes the care, prevention, and oral education of hospitalized patients. It originated in the United States, in 1901, when the first Department of Dentistry was structured at the Philadelphia General Hospital by the Dental Service Committee of the American Dental Association. In relation to Brazil, the exact origin is not known, but it is known that it started in the mid-20th century by institutions and professionals who were engaged in integrating oral and general care of hospitalized patients.^7^

In 2004, the Brazilian Association of Hospital Dentistry (ABRAOH) was created, which brought together dentists interested in the research, study, improvement, and dissemination of hospital dentistry.^6^ However, hospital dentistry was strengthened in 2005 when the Ministry of Health provided grants for residency programs in oral and maxillofacial surgery and traumatology at universities as part of the National Oral Health Policy – Brasil Sorridente.^5^

In 2008, Bill No. 2776/2008 was presented in the Chamber of Deputies, which provides for the obligation of reference dentists for Intensive Care Units.^1^ With the implementation of Ordinance GM No. 1032 of May 5, 2010,^9^ the Ministry of Health determined the inclusion of dental procedures in the Table of Procedures, Medicines, Orthoses and Prostheses, and Special Materials of the SUS for the care of PSN in hospitals, with the legitimacy of Hospital Dentistry.^9^

Resolution No. 7 of February 24, 2010,^10^ of the National Health Surveillance Agency established the minimum requirements for the operation of Intensive Care Units and stipulated that dental care must be guaranteed to patients in these units by their own or outsourced means (Section IV, Access to Assistance Resources, Article 18) and that dental care must be integrated with other care activities provided to the patient, being discussed jointly by the multiprofessional team (Section V, Work Processes, Article 23).^10^

**Figure 1:**
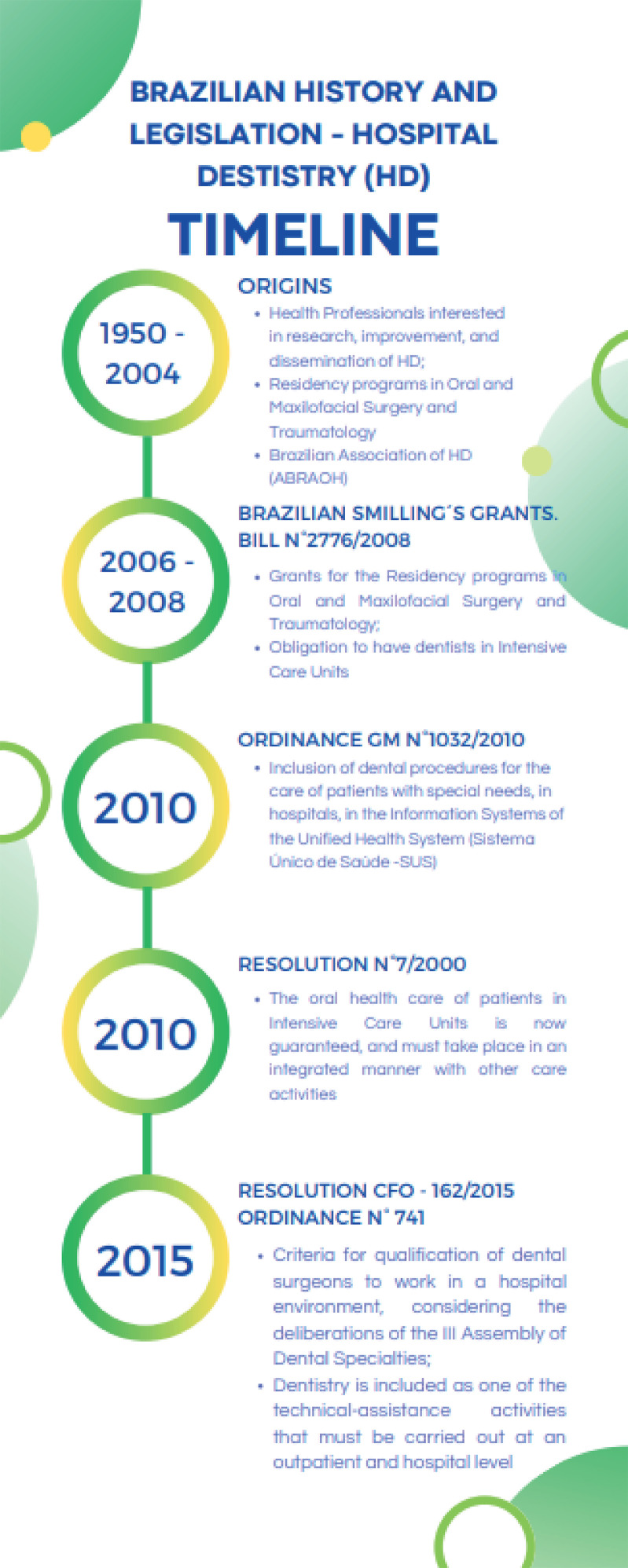
Timeline describing Brazilian history and legislation on the implantation of Hospital Dentistry.

Source: Hospital Information System (SIH). Tabnet DATASUS. In blue (health promotion and prevention), orange (Clinical procedures), gray (Surgical and diagnostic procedures) and yellow (Total procedures). The graphic curve of dental procedures performed is shown.

Resolution CFO-162 of 2015 of the Federal Council of Dentistry (CFO)^11^ recognized the practice of hospital dentistry by dentists, establishing the training criteria required for working in the hospital environment, considering the deliberation of the III National Assembly of Dental Specialties (ANEO), held on October 13 and 14, 2014, in São Paulo (SP).^11^ In addition, in accordance with Ordinance No. 741 of December 19, 2005, of the Ministry of Health,^12^ Dentistry is included as one of the technical-assistance activities that must be carried out on an outpatient and inpatient basis - routine and emergencies at the High Complexity Oncology Care Units (UNACON), the High Complexity Oncology Care Centers (CACON), and the High Complexity Oncology Reference Centers^12^ The timeline of hospital dentistry in Brazil is summarized in Figure 1.

Despite the importance of dental interventions in hospitals and public health policies determining the participation of oral health in the three levels of health care, the presence of dentists in hospital staff is still incipient. This study aimed to describe and analyze hospital dental care for PSN in Brazil, based on data from hospitals of the SUS.

## Methodology

The methodological study design was a descriptive and analytical cross-sectional observational investigation, based on secondary data from reports from the SUS (Sistema de Informações Hospitalares do SUS- SIH/SUS) from January 2010 to December 2018 from all Brazilian municipalities, which registered the total number of hospitalization authorizations with dental treatment for patients with special needs as the main procedure (*Total de Autorizações de Internação Hospitalar* – AIH).

The data were initially analyzed by descriptive statistics to characterize the sample, by absolute distributions, percentages, measures of central tendency, and dispersion (mean, median, minimum, maximum and standard deviation). To verify whether the regression residuals followed a normal distribution, the Kolmogorov-Smirnov test was applied. Then, multivariate analysis was performed to identify associations between the dependent and independent variables. Prevalence ratio (PR) values were obtained for each category of associated factors, considering a confidence interval of 95% and a statistical significance of 5%. The Statistical Package for Social Sciences (SPSS) software (IBM- SPSS, v.24, IBM, Chicago, USA) was used to perform the statistical analyses.^13^

Poisson regression was used to verify factors associated with dental treatment for PNS. Population size, region of the country, municipal HDI, oral health coverage in the Family Health Strategy, and number of dentists in hospitals were used as independent variables of the regression to predict the provision of dental care to PSN in a hospital environment adjusted for the number of SUS hospital beds per municipality.

The dependent variable total number of hospitalization authorizations with dental treatment for PSN as the main procedure (total AIH) was used as discrete. The independent variables were categorized in detail and presented in absolute (N) and relative (%) frequencies, namely: a) Population Size – up to 50 thousand inhabitants, 50,001 to 100 thousand inhabitants, and more than 100 thousand inhabitants; b) Country macro-region – North, Northeast, Southeast, South, Center-West; c) Municipal HDI^10^– Medium (between 0.500 and 0.799) and High or Very High (≥ 0.800); d) Gini index^11^ – ranging from 0 to 1; e) Oral health coverage in the Family Health Strategy – ranging from 0% to 100% of the municipality’s population; f) Number of dentists registered in a specialized hospital, general hospital, day hospital, emergency room of a general hospital (old) with SUS care in the municipality and number of SUS hospitalization beds per municipality.

The Municipal HDI and the Gini Index were obtained from the 2010 Demographic Census, released by the United Nations Development Program (P*rograma das Nações Unidas para o Desenvolvimento* - PNUD).^14^ Oral health coverage was provided by the Ministry of Health’s Primary Health platform (*Plataforma e-Gestor da Atenção Básica do Ministério da Saúde* – e-Gestor AB).^15^ The number of dentists and inpatient beds were obtained from the National Registry of Health Establishments in Brazil (*Cadastro Nacional dos Estabelecimentos de Saúde do Brasil-* CNES).^16^ Data on population size of each municipality were made available by the Brazilian Institute of Geography and Statistics (*Instituto Brasileiro de Geografia e Estatística* – IBGE).^17gl^

Furthermore, for the characterization and comparison of the relationships between Municipal HDI and quantitative variables total hospitalization authorizations and oral health coverage in the Family Health Strategy, the Spearman’s Rank Correlation Test with level of significance of α = 0.05 (5%) was used.

## Results

**Figure 2:**
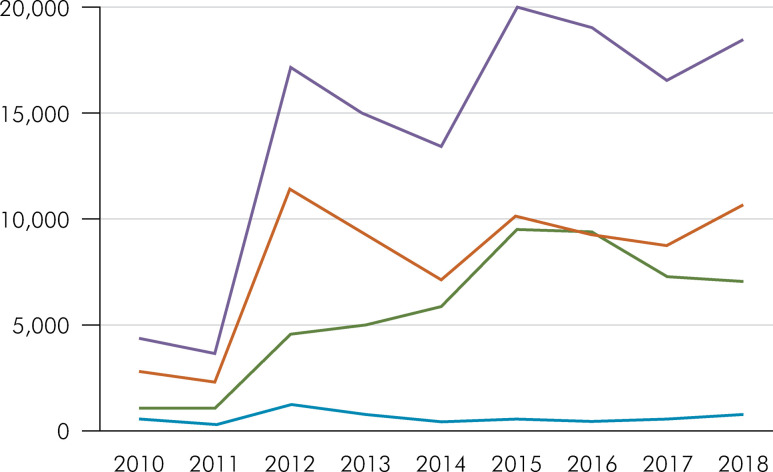
Total number of dental procedures performed on patients with special needs in a hospital environment by classification of procedures, Brazil, 2010–2018.

From 2010 to 2018, as shown in Figure 2, 127,691 dental procedures were performed in hospital units in PSN in 14,899 counties. Of these, 71,517 (56%) were clinical procedures, such as restorations, endodontic treatments, supra and subgingival scaling, among others. Surgical and diagnostic procedures accounted for 50,609 (39.6%), and a smaller portion, 5,565 (4.4%), were oral health promotion and prevention procedures.

As described in Table 1, of the 14,899 hospital admission authorizations (AIH) as main procedures in PSN, 13,220 (88.7%) were performed in municipalities with a population of over 100,000 inhabitants, 8,951 (60.1%) in municipalities in the Southeast region and 99.2% (14,777) with high or very high IDHM (Table 1).

**Table 1:** Number of authorizations for hospital admissions (AIH) with dental care for patients with special needs as main procedure, according to population size, macroregion, and municipal human development index, Brazil, 2010–2018.

Variables	Hospital admission authorization	
	n	%
Population size		
< 50,000 inhabitants	177	1.2
50,001–100,000 inhabitants	1,502	10.1
> 100,000 inhabitants	13,220	88.7
Macroregion		
Midwest	1,222	8.2
South	2,130	14.3
Southeast	8,951	60.1
Northeast	2,428	16.3
North	168	1.1
Municipal human development index		
Medium (between 0.500 and 0.799)	122	0.8
High or Very High (≥ 0.800)	14,777	99.2
Total	14,.899	100.0

Source: Hospital Information System (SIH). Tabnet DATASUS.

On average, the municipalities had 8.6 AIHs, 24.4% of the population had oral health coverage in the Family Health Strategy, had 57.4 dentists in Hospitals, except CBO (Brazilian Classification of Occupations) for oral and maxillofacial traumatologists, and 19.5 Oral and Maxillofacial Traumatology dentists. The number of hospital beds in the analyzed municipalities varied between 31 and 15,666 (Table 2).

**Table 2:** Descriptive analysis of the total authorization for hospital admission (AIH), with dental care for patients with special needs as the main procedure, GINI index, oral health coverage in the family health strategy, number of dentists and number of hospital beds, Brazil, 2010–2018.

Variables	Median	Minimum	Maximum	Average	Standard deviation
Total AIH	33	1.0	327.0	8.6	9.7
Gini index	1	0.0	1.0	0.8	0.4
Oral health coverage in the family health strategy (CD number per population)	16.9	0.0	100.0	24.4	23.7
Dentists in Hospital by municipality (except Oral and Maxillofacial Traumatologist CBO)	3	0.0	499.0	57.4	93.7
Dentists in Hospital by municipality (only Oral and Maxillofacial Traumatologist CBO)	1	0.0	121.0	19.5	27.4
Hospital beds by municipality	1352	31.0	15666.0	3048.0	4144.5

Source: IBGE, Hospital Information System (SIH) and National Register of Health Establishments in Brazil (CNES).

The Kolmogorov-Smirnov test indicated that the regression residuals did not follow a normal distribution (p < 0.001).

Population size, region of the country, and the number of dentists in hospitals were associated with dental care to PSN in a hospital environment, adjusted for the number of hospital beds. Municipalities with more than 100,000 inhabitants (PR = 2,593) performed more visits compared to smaller ones. Municipalities in the Midwest (PR = 5,117) and Southeast (RP = 4,443) regions provided more care than the North region. The cities that had more dentists in hospitals (except for oral and maxillofacial traumatologist CBO) (PR = 1,002) provided more care to PSN. The opposite occurred in municipalities with more oral and maxillofacial dentists (PR = 0.983), in which the number of consultations was lower (Table 3).

**Table 3:** Poisson regression of factors associated with dental care for patients with special needs in a hospital environment, Brazil, 2010–2018.

Variables	B	Standard error	p-value	Prevalence ratio	95%CI	
					Lower	Upper
Population size						
< 50,000 inhabitants	-	-	-	1	-	-
50,001–100,000 inhabitants	0.770	0.1298	< 0.001*	2,159	1,674	2,785
> 100.000 inhabitants	0.953	0.1298	< 0.001*	2,593	2,011	3,344
Macroregion						
North	-	-	-	1	-	-
Northeast	0.967	0.1107	< 0.001*	2,631	2,118	3,269
Southeast	1,491	0.1101	< 0.001*	4,443	3,581	5,512
South	1,172	0.1129	< 0.001*	3,228	2,587	4,027
Midwest	1,633	0.1102	< 0.001*	5,117	4,123	6,351
Municipal human development index						
High or very high (≥ 0.800)	-	-	-	-	-	-
Medium (between 0.500 and 0.799)	-0.148	0.0941	0.116	0.863	0.717	1,037
Oral health coverage in the family health strategy	0.001	0.0006	0.058	1.001	1.000	1,002
Dental surgeons in hospital (except Oral and Maxillofacial Traumatologist CBO)	0.002	0.0002	< 0.001*	1.002	1.002	1,003
Dental surgeons in hospital (only Oral and Maxillofacial Traumatologist CBO)	-0.017	0.0008	< 0.001*	0.983	0.981	0.984

95%CI: 95% confidence interval; *Statistically significant (p < 0.05).

According to Table 4, the Spearman’s correlation test showed that there is no statistical evidence to reject the null hypothesis of the correlation between municipal HDI and total hospitalization authorizations, that is, there is no correlation between the two variables. For the variables municipal HDI and oral health coverage in the Family Health Strategy, the null hypothesis that there is no correlation between the two variables was rejected, but the correlation coefficient was very close to 0, indicating a very weak, almost non-existent correlation.

**Table 4:** Spearman rank correlation between municipal Human Development Index (HDI) and Total Authorizations for Hospital Admission (AIH) and oral health coverage in the family health strategy (CoverageSBSF).

Variable	p-value	Test’s decicion	Coeffcient p
Total AIH and municipal human development index	0.4387	HO is not rejected	
Municipal human development index and oral health coverage in the family health strategy	< 0.001	HO is rejected	-0.074
Total AIH and oral health coverage in the family health strategy	< 0.001	HO is rejected	-0.2

## Discussion

The presence of dentists in hospitals is broad, being indispensable, since they are often responsible for the control of hospital receipts, which helps reduce hospitalization time, in addition to acting in emergencies (pain, hemorrhages, and fractures) and diagnosis and treatment of oral lesions associated with systemic diseases. Thus, dentists are important in several medical fields, including oncology, hematology, cardiology, dermatology, psychiatry, intensive care, endocrinology, organ and tissue transplantation, neurology, rheumatology, infectious diseases and nephrology.^6,7,18-24^ In addition, as mentioned, the presence of dentists in multidisciplinary hospital teams is already foreseen and legally encouraged in Brazil.

Although there is ample evidence in the literature of the importance of the presence of the dentist in highly complex multidisciplinary teams^6,7,18-24^and hospital dentistry is already politically recognized as an important axis for dentistry in promoting oral health,^1-6^ the presence of this professional in the hospital care of patients with special needs in the SUS is still negligible, given that less than 130,000 dental procedures were performed in a hospital setting in PSN in a period of 8 years.

Although the specialty of Oral and Maxillofacial Surgery and Traumatology was the structuring pillar of hospital dentistry,^6^ most of the procedures in a hospital setting in PSN were clinical procedures, such as restorations, endodontic treatments, supra and subgingival scaling, among others. In other countries, according to a comprehensive review that included experiences from countries in Europe, Asia, and Oceania, it was observed that there is a demand for invasive dental interventions, including periodontal, restorative, surgical, and endodontic treatment. However, the insertion of the dentist in the hospital routine is incipient and essentially linked to Oral and Maxillofacial Surgery and Traumatology.^25,26^ This scenario differs from the Brazilian context, where we observed that most interventions were clinical procedures rather that surgical and diagnostic procedures.

The Poisson regression results, when jointly evaluating several independent variables, pointed to an inequality in hospital care for PSN in different municipalities and macro-regions, with higher access in more populous municipalities and with better socioeconomic conditions. Furthermore, when analyzed separately, there was a correlation between municipal HDI and oral health coverage under the Family Health Strategy, which reinforces previous observations. However, the correlation coefficient was very close to 0, indicating a very weak, almost nonexistent correlation. This fact can be explained by the sample size (n = 14.899), so that even very weak correlations are statistically significant. On the other hand, no correlation was found between municipal HDI and total number of hospital admission authorizations (when analyzed separately).

With regard to multidisciplinary health care, statistical analysis in the last decade and previous periods indicate that there in no horizontal equity in access to medical appointments in most states, with a pattern that favors people in better socioeconomic conditions over individuals with social vulnerabilities, especially PSN.^28,29^ This scenario, as observed in the results of this study, also seems to occur in hospital dentistry and in indicators of primary and secondary dental care, since there was a statistical difference between municipal HDI and Family Health Strategy oral health coverage variables.

If, on the one hand, larger and better structured municipalities are expected to implement highly complex health services beforehand, on the other hand, it is important that the others have reference to guarantee more complex care. An example of this was the Living Without Limits Program (*Viver sem Limites Programa)*, which provided funding for Dental Specialty Centers (CEO), with professionals working 40 hours a week with PSN and referral hospitals, ensuring comprehensive care through a polyarchy organization of the oral health care network.^27^

Regarding health promotion and prevention actions, 5,565 procedures were registered over the 8 years, corresponding to only 4.4% of the total. In the context of the COVID-19 pandemic, clinical procedures were also the majority. As observed by a literature review on hospital dentistry and dental care for PSN in Brazil, only dental urgencies and emergencies should be performed during the pandemic to reduce the formation of aerosols from procedures and restrict the flow of patients to dental offices.^28^ The procedures included control of oral bleeding, drainage of cellulitis or dental abscesses, major oral surgery for traumas involving facial bones with potential involvement of the patient’s airways, procedures for resolving dental or soft tissue pain, odontogenic infections, oral bleeding, and oral necrosis.^28,29^

Finally, this study had limitations because of the use of secondary data. Although data were not collected directly by the research team, it was registered with strict control and calibration criteria in the databases. The dental procedures recorded in the SIH/SUS allow a very large sample covering all the municipalities that register their productions, but rigorous registration of all data by all the teams without irregularities throughout the historical series cannot be confirmed, which may mean the loss of relevant information for the purpose of the research.

## Conclusion

This study aimed to describe and analyze the dental procedures performed in PSN in hospitals of the SUS.

Although the importance of hospital dentistry in the SUS for an integral and humanized rehabilitation of PSN has been recognized, it is still not well established and structured in health services and poorly distributed among different municipalities, reinforcing inequalities in access to health care and highlighting the need for greater action to promote and expand the presence of dentists in multidisciplinary teams in high hospital complexity.
